# Fighting Viral Infections and Virus-Driven Tumors with Cytotoxic CD4^+^ T Cells

**DOI:** 10.3389/fimmu.2017.00197

**Published:** 2017-02-27

**Authors:** Elena Muraro, Anna Merlo, Debora Martorelli, Michela Cangemi, Silvia Dalla Santa, Riccardo Dolcetti, Antonio Rosato

**Affiliations:** ^1^Immunopathology and Cancer Biomarkers, Traslational Research Department, IRCCS, C.R.O. National Cancer Institute, Aviano, Pordenone, Italy; ^2^Department of Immunology and Blood Transfusions, San Bortolo Hospital, Vicenza, Italy; ^3^Istituto Oncologico Veneto IOV-IRCCS, Padova, Italy; ^4^Translational Research Institute, University of Queensland Diamantina Institute, Brisbane, QLD, Australia; ^5^Department of Surgery, Oncology and Gastroenterology, Oncology and Immunology Section, University of Padova, Padova, Italy

**Keywords:** CD4^+^ T cells, cytotoxicity, viral infection, virus-driven tumors, immunotherapy

## Abstract

CD4^+^ T cells have been and are still largely regarded as the orchestrators of immune responses, being able to differentiate into distinct T helper cell populations based on differentiation signals, transcription factor expression, cytokine secretion, and specific functions. Nonetheless, a growing body of evidence indicates that CD4^+^ T cells can also exert a direct effector activity, which depends on intrinsic cytotoxic properties acquired and carried out along with the evolution of several pathogenic infections. The relevant role of CD4^+^ T cell lytic features in the control of such infectious conditions also leads to their exploitation as a new immunotherapeutic approach. This review aims at summarizing currently available data about functional and therapeutic relevance of cytotoxic CD4^+^ T cells in the context of viral infections and virus-driven tumors.

## CD4^+^ T Cell Lineage Development and Plasticity

Thymic development of T cells clearly distinguishes two different fates for the MHC class II-restricted CD4^+^ T helper (Th) and the MHC class I-restricted CD8^+^ cytotoxic T cell lineages (1). However, it is now widely believed that CD4^+^ T cells can also exert cytolytic activity beside the helper function, as demonstrated by *in vitro* (2, 3) and *in vivo* evidence (4–9).

The main role of CD4^+^ T cells is to indirectly orchestrate the immune response by differentiating into distinct Th cell populations. These subsets are characterized by specific differentiation signals, expression of distinct master transcription factors, secretion of signature cytokines, and specific functions (10–12). The first functional diversification proposed, identified, and separated Th1 from Th2. Th1 cells are induced by interleukin (IL)-12, express T-bet, and target intracellular pathogens through the release of interferon (IFN)-γ. Conversely, Th2 lymphocytes are stimulated by IL-4, are characterized by GATA-3 expression and IL-4 production, and play a critical role in fighting extracellular parasites (13–15). During the years, several other functionally distinct subsets of helper CD4^+^ T cells have been identified and characterized. Th17 cells control fungi and extracellular bacteria through the release of IL-17 and IL-22 (16, 17). Follicular helper T cells reside in B cell follicles and are essential for the generation of B cell memory (18, 19). Th9 are involved in allergic asthma (20), whereas Th22 act in skin immune defense (21). Finally, regulatory T cells (Treg) represent an heterogeneous population that plays a key role in mediating peripheral tolerance and include naturally occurring Treg, Type 1 Treg, and Th3 cells (22–24). The modulatory activities of Type 1 Treg are mainly mediated by TGF-β, but seem to depend also on specific cell-to-cell interactions. This interplay results in the selective killing of myeloid antigen-presenting cells (APC) through a mechanism depending on granzyme B and perforin (HLA class I-mediated) (25), suggesting a direct activity of CD4^+^ T lymphocytes against target cells.

Similarly, CD4^+^ cytotoxic T lymphocytes (CD4^+^ CTL) have been described for their direct contribution to control infections and malignancies as being capable of lysing class II-expressing targets (10). Initially considered as an *in vitro* artifact (2, 3), CD4^+^ CTL have been isolated in mice and humans in various pathologic conditions, including viral infections [human immunodeficiency virus (HIV) 1, influenza virus, cytomegalovirus (CMV), and Epstein–Barr virus (EBV)], autoimmune and autoinflammatory diseases (rheumatoid arthritis, ankylosing spondylitis), and malignancies (B cell chronic lymphocytic leukemia) (5–9, 26), as well as after vaccination (27, 28). While in healthy individuals the percentage of CD4^+^ CTL hardly exceeds 2% of total peripheral CD4^+^ T cells, they are markedly increased in the presence of chronic viral infections, reaching in some HIV-1-infected individuals up to 50% of the CD4^+^ T cells and exhibiting a clear cytotoxic potential against viral antigens (6, 26, 29, 30). *In vitro* experiments demonstrated that the cytotoxic ability of these effectors is not conferred by soluble mediators, but rather by a direct cell-to-cell contact (28). Originally assimilated to the more classical CD4^+^ T cells, CD4^+^ CTL display distinct surface markers and functional properties that relate them to Ag-experienced end-stage differentiated CD4^+^ T cells (6).

Intriguingly, it is becoming increasingly clear that belonging to the above-described differentiation lineages is not an irreversible program in CD4^+^ T cell development. Indeed, recent evidence indicates that some CD4^+^ T cells maintain a certain degree of plasticity, which allows the acquisition of characteristics of alternative lineages upon antigen restimulation (24, 31). T-cell stability and plasticity are regulated by different factors such as cellular conditions (cytokines and costimulatory molecules), transcriptional circuitries, and chromatin modifications (32). Since the expression of a master regulator may be transient or dynamic, it would be more appropriate to consider the levels, ratios, and context of expression rather than the mere presence/absence of transcription factors as they could change during the course of immune stimulation (11). Moreover, the interplay between lineage-specifying transcription factors, including T-bet (Th1), GATA-3 (Th2), ROR-γt (Th17), and FoxP3 (Treg), which are frequently co-expressed, contributes to determine the final outcome of the gene expression profile of CD4^+^ T cells (33). T-cell differentiation and plasticity are also controlled by several microRNA (miRNA), the “immunomiRs,” involved in T cell thymic development (miR-181a and miR-150), activation (miR-21, miR-155, and miR-17~92), or functional differentiation (miR-126 and miR-146a) (34, 35). Epigenetic processes are also involved in T-cell plasticity because they facilitate hereditable and stable programs of gene expression while preserving the possibility to be modified in response to environmental changes. For example, DNA methylation and histone deacetylation dampen the expression of both Th1- and Th2-specific cytokines (36) and cytosine methylation controls CD4 expression, which is silenced in CD8^+^ T cells and stably expressed in CD4^+^ T cells (37). The notion of CD4^+^ T cell plasticity, which clarifies that CD4^+^ T cell differentiation states are not definitive (12), challenges the concept of irrevocable CD4 *versus* CD8 lineage commitment (38). This concept legitimizes the hypothesis that, in response to chronic or strong antigen stimulation, mature CD4^+^ T cells can be able to switch off their expression of the helper T cells master regulators and differentiate into functional CTL (39).

## Transcriptional Factors Involved in the Acquisition of Cytolytic Ability by CD4^+^ T Cells

**Figure 1:**
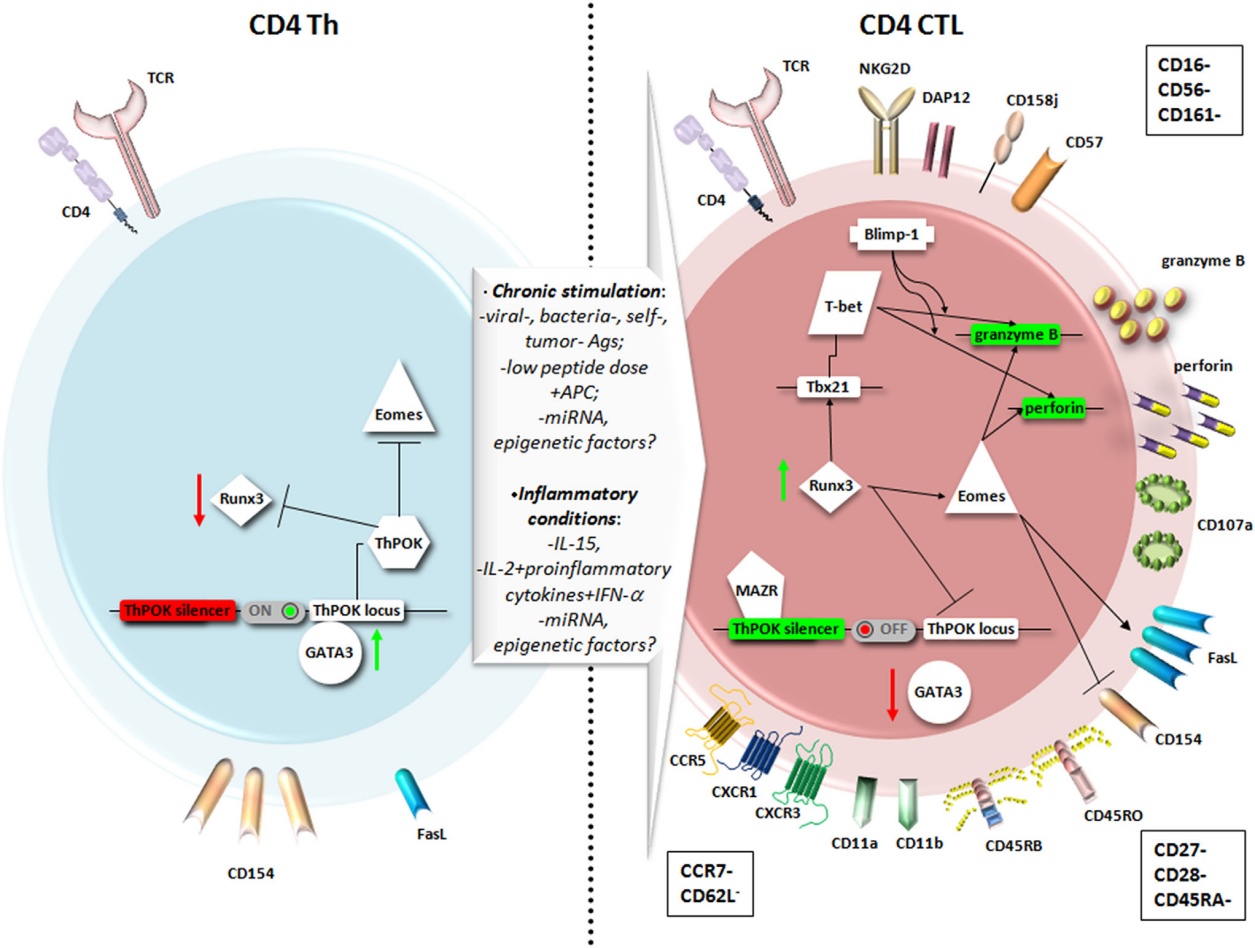
**Main phenotypic features and transcriptional pathways involved in the differentiation of CD4^+^ T helper (Th) cells into CD4^+^ cytotoxic T lymphocytes (CD4^+^ CTL)**. A chronic stimulation in the presence of inflammatory conditions may favor the expression of genes responsible for a cytotoxic T lymphocytes (CTL) fate for CD4^+^ T cells as Runt-related transcription factor 3 (Runx3) and Eomesodermin (Eomes), at the expense of genes usually expressed by CD4^+^ Th, as Th-inducing BTB/POZ domain-containing Kruppel-like zinc-finger transcription factor (ThPOK) and GATA3. Therefore, CD4^+^ CTL express higher levels of Fas ligand (FasL) compared to CD4^+^ Th, cytotoxic granules with perforin and granzyme, and the degranulation marker CD107a. The particular phenotype reflects a highly differentiated memory cell, expressing typical NK markers, and equipped to migrate to peripheral tissues through chemokine receptors.

During thymic development, the CD4^+^ *versus* CD8^+^ lineage commitment from the common CD4^+^CD8^+^ thymocyte precursors is critically controlled by key transcription factors. The Th-inducing BTB/POZ domain-containing Kruppel-like zinc-finger transcription factor (ThPOK) suppresses the cytolytic program in MHC class II-restricted CD4^+^ thymocytes (Figure 1, left panel). On the contrary, in MHC class I-restricted precursor cells, the Runt-related transcription factor 3 (Runx3) opposes ThPOK and promotes the lineage commitment of CD8^+^ cytolytic T lymphocytes (CD8^+^ CTL) (40). It is important to underline that this functional programming coincides but does not depend on the MHC restriction or expression of the co-receptor CD4 or CD8αβ, but it is rather controlled by the activity of these key transcription factors (39).

On this assumption, it was recently demonstrated that through a unique mechanism of plasticity at postthymic level, mature CD4^+^ Th cells can convert themselves to cytotoxic MHC class II-restricted effectors at the expense of becoming inflammatory or immunosuppressive cells, turning off ThPOK expression (39, 40), as shown in the right panel of Figure 1. The authors identified in the ThPOK silencer the transcriptional switch that terminates ThPOK transcription, which in turn favors the derepression of the CTL program in mature CD4^+^ effector cells, leading to the acquisition of the cytolytic potential (39). The repressive activity of the ThPOK silencer, which is suppressed by the same ThPOK, is activated by the zinc-finger transcription factor MAZR (also called PATZ1 or Zfp278) (41) that, in turn, functions as a negative regulator of ThPOK (39). MAZR seems to act by directly binding to ThPOK silencer, even though the involvement of other transcription factors cannot be excluded (41) (Figure 1, right panel).

Studies performed in the gut mucosa revealed that most CD4^+^ intraepithelial lymphocytes show modest expression of ThPOK, while expressing high levels of Runx3. These cells lost their differentiation into the Th17 subset and their colitogenic potential, avoiding the excessive activation of an inflammatory response in the intestine (42). Runx3 may thus intervene in the suppression of both ThPOK expression and Th17 differentiation, whereas it induces an increased expression of Tbx21 mRNA, which encodes for T-bet (42). This transcription factor, together with Eomesodermin also known as T-box brain protein 2, Trb2, encoded by the EOMES gene, critically controls the effector functions of CTLs. In particular, *in vitro* experiments demonstrated that the introduction of Eomesodermin alone is sufficient to convert the functions of fully differentiated Th cells toward those of CTL. Ectopic expression of this transcription factor leads to a decrease in CD154 (namely the CD40 ligand) upregulation, is pivotal for helper function (Figure 1, left panel), and elicits perforin expression and Fas ligand (FasL) upregulation (Figure 1, right panel). Interestingly, the cytolytic activity of Eomesodermin transfectants appeared more efficient than that of perforin-transfected cells, indicating that Eomesodermin may play a critical role in the activation of granule exocytosis pathway (43). Eomesodermin expression is strictly regulated by Runx3 and ThPOK, which are, respectively, responsible for the induction and the suppression of its expression in CD4^+^ T cells (43) (Figure 1, right and left panels, respectively). The enforced expression of Eomesodermin in CD4^+^ T cells induces a 10–100 times higher expression of cytotoxic genes such as perforin and granzyme B, if compared to naive CD4^+^ T cells, even if much lower than in CD8^+^ effector cells (44). It is still not clear whether the acquisition of cytotoxic gene expression implies the cessation of ThPOK expression or antagonism of its function (37). In Th2 cells, Eomesodermin, which is highly expressed in memory cells but downregulated in IL-5-producing effectors, interacts with GATA-3 preventing its binding to IL-5 promoter (45). GATA-3 is a crucial transcription factor already in early CD4^+^ T cell lineage differentiation acting upstream of ThPOK (1, 46), and it is mutually expressed with Runx3 (40). Since GATA-3 binds to ThPOK locus (Figure 1, left panel) and likely relieves Runx-dependent ThPOK repression (47), its down modulation could be also involved in the CD4^+^ T cells acquisition of cytolytic potential controlled by Runx3 and Eomesodermin expression (Figure 1, right panel).

CD4^+^ CTL appear thus distinct from Th1 cells, even though their differentiation could follow a Th1 developmental pathway, with IL-12 contributing to granzyme B and perforin induction (28, 48). In this context, in a mouse model of melanoma, the CD134 (OX40) and CD137 (4-1BB) costimulation as immunotherapy in the presence of IL-2, imprinted a cytotoxic phenotype relying on Eomesodermin, on both Ag-specific and bystander CD4^+^ Th1 cells (49). Similarly, CD4^+^ T cells with cytotoxic potential are specifically induced at the site of infection during influenza virus infection. The development of these CD4^+^ CTL seems to depend on the cooperation of the STAT2-dependent type I IFN signaling and the IL-2/IL-2 receptor-α pathway, which induce the T-bet and Blimp-1 transcription factors (50). The transcriptional repressor Blimp-1 favors the binding of T-bet to the promoters of cytolytic genes as granzyme B and perforin, independently of its DNA binding/repressor activity. Blimp-1 likely acts also by repressing other repressors of the granzyme B and perforin genes, such as miRNA targeting these genes, thus favoring their expression (28, 50) (Figure 1, right panel).

## Differentiation Markers, Localization and Activation of CD4^+^ CTL

The acquisition of cytotoxic potential in CD4^+^ T cells is probably dependent on a postthymic differentiation process characterized by the sequential attainment of lytic granules with granzymes and perforin and the parallel loss of CD27 and CD28 surface expression that identify them as highly differentiated, end-stage cells resulting from chronic stimulation (6, 26, 51). Interestingly, the ability to acquire cytotoxic activity by conventional CD4^+^ T cells seems to be influenced by both Treg and CD8^+^ T cells. Indeed, in a transgenic mouse model, Treg depletion significantly increased the production of cytokines and granzyme B in virus-specific CD4^+^ T cells, but did not affect the CD4^+^ T-cell-mediated MHC class II-restricted cytotoxicity, which was clearly enhanced only after CD8^+^ T cells depletion (52). This means that CD4^+^ CTL could play a critical role in chronic infection when cytotoxic CD8^+^ T cells become functionally exhausted (52).

In different infections, virus-specific CD4^+^ T cells have been described with distinct differentiation phenotypes, with CMV-specific CD4^+^ T cells more differentiated than CD4^+^ T cells recognizing influenza virus, hepatitis C virus (HCV), EBV, and HIV-1 (26). Indeed, *in vivo* models of influenza revealed the presence of CD4^+^ T cell-mediated cytotoxicity also in response to acute infection and showed the presence of both CD27^+^ and CD27^−^ CD4^+^ granzyme^+^ T cells in the lung, while chronic infection with γ-herpes virus may further differentiate CD4^+^ CTL to CD27^−^ cells (10). CD4^+^ CTL generated in mouse models also show an increased expression of the CD43 activation marker (10). The high expression of the CD11a and CD11b integrins, and the RO and RB CD45 isoforms (but not the RA) usually observed in CD4^+^ CTL, further supports their belonging to the memory pool of CD4^+^ T cells (28, 30) (Figure 1, right panel). Usually, CD4^+^ CTL do not show activation or proliferation markers, being mainly CD38^−^, CD69^−^, HLA-DR^−^, and present high levels of Bcl-2 and a weak staining for the active proliferation marker Ki-67 (6, 39, 51), suggesting a non-activated phenotype and very low turnover as long-lived cells, at steady state. Notably, Mucida and colleagues reported a higher expression of CD69 in CD4^+^ CTL compared to CD4^+^ Th cells and related this feature to strong and repeated activation signals (39).

As shown in the right panel of the Figure 1, commonly expressed markers in CD4^+^ CTL also include CD57 and NK receptors, such as the killer immunoglobulin-like receptor CD158j, KARAP/DAP12 (killer cell activating receptor-associated protein/DNAX activating protein of 12 kDa), and the killer lectin receptor NKG2D (but NK/NKT-specific markers as CD16, CD56, and CD161 are absent) (51), as well as the degranulation marker CD107a (LAMP-1) (39). Another typical marker predominantly expressed on activated CD8^+^ T cells and NK/NKT cells was observed in a small fraction of CD4^+^ T cells: the cytotoxicity-related, MHC class I-restricted, T cell-associated molecule CRTAM. The unique population of CRTAM^+^CD4^+^ T cells was observed in mucosal tissues and inflammatory sites and may likely represent precursors of CD4^+^ CTL (53).

Conversely, CD4^+^ CTL lack CCR7 and CD62L expression, which precludes their migration to lymph nodes, although these cells are equipped to migrate to peripheral tissues through chemokine receptors as CCR5, CXCR3, and CX3CR1 (51) (Figure 1, right panel).

Interestingly, phenotypic variations of CD4^+^ CTL can be observed also according to the anatomic sites in which these cells develop. For example, the receptor for epithelial cadherin CD103 appears highly expressed only in CD4^+^ CTL generated in the lungs of influenza-infected mice (54). The environment of the lung is probably better suited for CD4^+^ CTL differentiation and activity, if compared to draining lymph nodes where CD4^+^ T cells do not express markers of cytolytic potential (28, 50). Similarly, the differentiation in cytotoxic effectors could also take place for CD4^+^ T cells migrating to the intestinal tissue and particularly to the intraepithelial compartment (42). The road map favoring this differentiation in the gut is different from that available in the setting of the thymus for CD8^+^ CTL and may be probably related to the presence of commensal microflora, as CD4^+^ CTL are absent in germ-free mice (38, 39). Both the lungs and gut are thoroughly exposed to environmental cues, which favor immune activation and chronic stimulation of lymphocytes, key factors for CD4^+^ CTL differentiation and activation, together with inflammatory conditions (26, 30, 40) (Figure 1).

The aforementioned environmental conditions, in which CD4^+^ CTL usually develop, include several cytokines directly responsible for the acquisition of cytolytic potential by CD4^+^ T cells. IL-15 is one of the candidates as it is present at abnormally elevated levels in several diseases showing increased numbers of CD4^+^ CTL, including rheumatoid arthritis and HIV-1 infection (6). In agreement with this observation, *in vitro* short-term incubation with IL-15 increased the number of CD4^+^ T cells expressing cytotoxic markers in PBMCs of both healthy donors and HIV-1-infected individuals (6). At steady state, CD4^+^ CTL appear immunologically quiescent. In addition to inflammatory conditions, their activation also requires the simultaneous stimulation with cognate antigen at low doses in the presence of APC (39, 55). Beyond IL-15, IL-2 also seems to play a pivotal role in the induction of cytotoxicity ability in CD4^+^ T cells. Indeed, strong IL-2 signals acting through Jak3 and STAT5 are necessary to induce *in vitro* granzyme B and perforin in CD4^+^ T cells (56). In addition, higher amounts of the type I IFN IFN-α and pro-inflammatory cytokines IL-6 and TNF-α, extend IL-2 signaling and optimize CD4^+^ CTL generation (28). Also IL-12 may contribute to this process by increasing the expression of perforin and granzyme B through the activation of STAT4, whereas IL-4 activates STAT6 and inhibits the cytotoxic activity of *in vitro* generated CD4^+^ CTL (54).

## Mechanisms of Action Responsible for the Cytolytic Activity of CD4^+^ CTL

The cytolytic activity of CD4^+^ CTL is mainly mediated by two major mechanisms of action: the exocytosis of cytotoxic granules, as perforin, granzyme B, and granulysin, and the Fas-dependent pathway. Both require a cell–cell direct interplay and T-cell receptor (TCR)/MHC/peptide interaction, which trigger cell degranulation in the first case and FasL upregulation in the second pathway (30, 51). The two mechanisms are not mutually exclusive, but rather appear to depend on external stimuli or environmental circumstances. High levels of peptides and absence of IL-2 stimulate the Fas:FasL mechanism, whereas low peptide doses and addition of exogenous IL-2 are required to induce perforin-mediated cytotoxicity (10). In the majority of cases, CD4^+^ CTL probably use the perforin-dependent mechanism to induce apoptosis in target cells, since these effector T cells develop mainly in the presence of IL-2 and low peptide doses (26, 51, 53). Perforin and granzyme B pathways are in some cases, as during influenza A virus (IAV) infection, the only cell killing mechanisms used by CD4^+^ CTL (28). The two mechanisms of action seem to be differentially employed depending on CD4^+^ CTL functional role. In particular, the Fas:FasL pathway is mainly implicated in CD4^+^ CTL ability to downregulate the immune response. Indeed, antigen-presenting B cells express high levels of FasL on their surface and are particularly sensitive to the Fas-mediated cytolytic pathway. On the contrary, perforin- and granzyme B-mediated cytotoxicity is involved in response to several viruses, as HIV-1, CMV, EBV, HSV, and influenza, and against B cell lymphocytic leukemia. On the other hand, granulysin is primarily used in case of mycobacterial and fungal infections (10). However, in all these circumstances, CD4^+^ CTL can also use Fas:FasL as a compensatory mechanism when IL-2 is limiting (48).

A few papers described other cytotoxicity mechanisms potentially employed by CD4^+^ CTL, including the TNF-related apoptosis-inducing ligand pathway (57), and the binding between CD154 (CD40L) expressed on T cells and CD40 on target cell surface. The latter may mediate cytolytic activity after immunization with a recombinant protein antigen associated to a TLR4 agonist (GLA-SE) (27).

To date, the overall functional activity of CD4^+^ CTL has been considered markedly weaker than the cytolytic capacity of CD8^+^ T cells. Several factors may underlie this assumption. Indeed, MHC class II is expressed by half of target cells, and the functional avidity of CD8^+^ T cells is 1,500-fold higher than that of CD4^+^ T cells. Thus, when adjusting CD4-mediated cytotoxicity for effector:target ratio, precise specificities, and functional avidities, the only difference between CD4^+^ and CD8^+^ T cells is the slightly delayed killing kinetics of CD4^+^ CTL (58). Interestingly, when measuring secreted amounts of granzyme B through ELISpot or ELISA assays, there are no relevant differences between activated memory CD4^+^ T cells and memory CD8^+^ T cells, thus demonstrating that CD4^+^ T cells can be major sources of extracellular granzyme B (59).

## Cytolytic CD4^+^ T Cells and Viral Immunity

**Figure 2:**
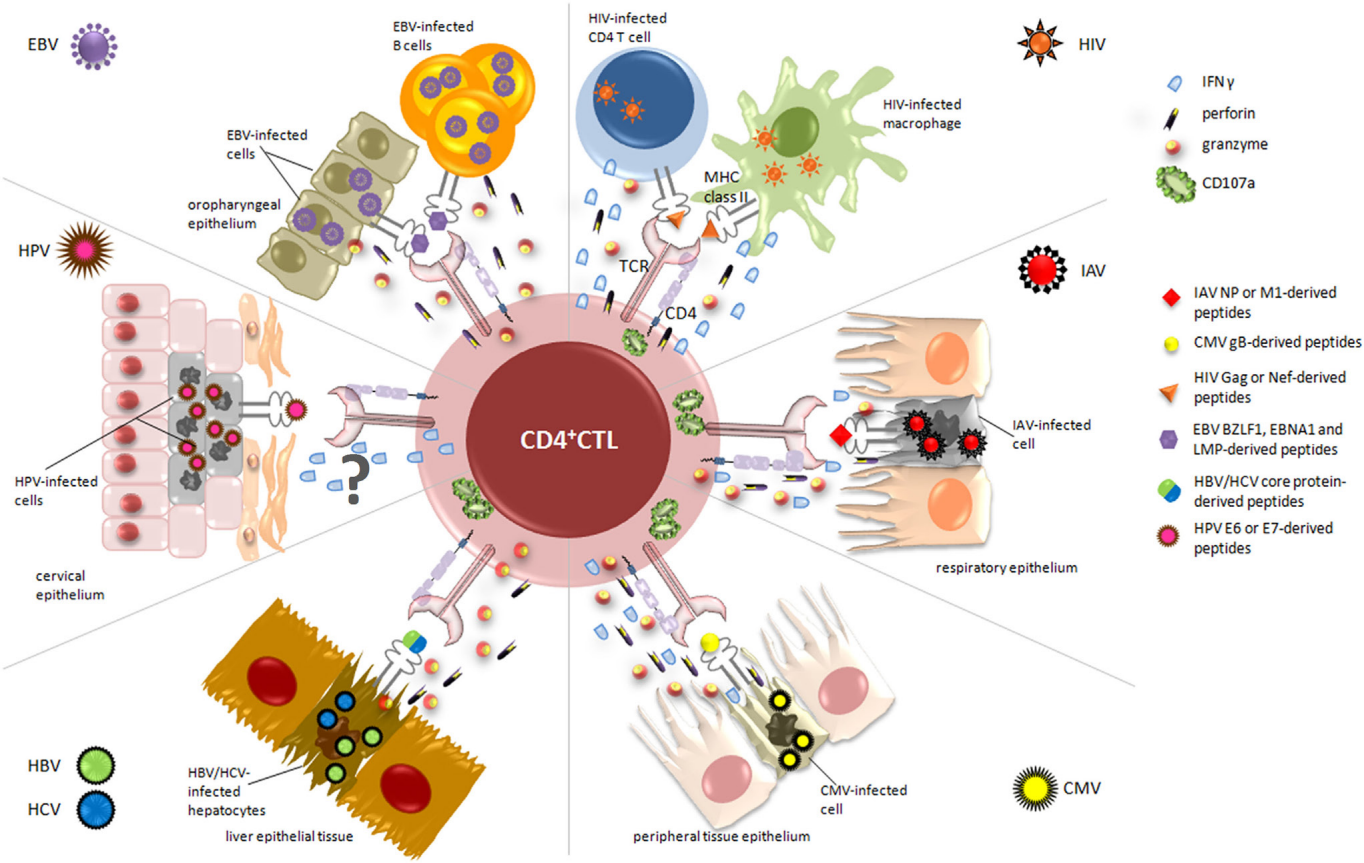
**Relevant cytotoxic mechanisms exerted by CD4^+^ cytotoxic T lymphocytes (CD4^+^ CTL) against virus-infected cells**. During viral infections, activated CD4^+^ CTL are able to specifically kill infected/dying targets, through the recognition of virus-derived peptides presented on MHC class II molecules. The figure displays the essential target cells from different organs and tissues, depending on viral tropism (i.e., cervical, respiratory and oropharyngeal epithelium, liver tissue), and highlights the main cytolytic granules and cytokines secreted by CD4^+^ CTL in response to each virus. Upon recognition of CD4-specific T cell epitopes, derived from immunodominant viral proteins such as nucleoprotein (NP) for IAV, lytic and latent proteins for Epstein–Barr virus (EBV), and E6/E7 for human papillomavirus (HPV), CD4^+^ CTL are able to kill infected cells through the release of high amounts of granzyme A and B, perforin, and interferon (IFN)γ and the degranulation of CD107a. The mechanism of action by which CD4^+^ CTL exert their cytotoxic activity against HPV-infected cells is still uncertain and requires further analysis.

Overall, CD4^+^ CTL appear to be involved in the recognition and the elimination of virus-infected cells in the manifold response activated by the immune system during viral infections. Accordingly, in the following sections, we described the contribution of CD4^+^ CTL to the immune response against different viruses. As summarized in Figure 2, for each virus, we reviewed the current literature mainly with respect to the antigens recognized by these effectors, the selected tropism for virus-infected target cells, the mechanisms of lysis differentially involved, and, when possible, vaccinations and immunotherapeutic strategies exploiting CD4^+^ CTL responses (Table 1).

**Table 1:** **Vaccinations and immunotherapeutic approaches exploiting cytotoxic CD4^+^ T cell responses**.

Virus	Host	Vaccinations and immunotherapeutic strategies	Reference
IAV	Human	Epitope-based (IAV-derived CD8^+^ and CD4^+^ T cell epitopes) universal influenza vaccine	(60)

CMV	Human, *in vitro* model	Recombinant CMV glycoprotein B subunit vaccine	(61)
	Mouse	MCMV peptide vaccination in immunocompetent mice	(62)
	Mouse	TCR transgenic mice able to recognize a MCMV-specific CD4^+^ T cell epitope within M25 protein	(63)

HIV	Rhesus macaques	DNA vaccination from *env-* and *nef-*deleted simian-human immunodeficiency virus	(64)
	Human	Phase III RV144 Thai trial; CD4^+^ T cell responses against the V2 region of the envelope protein	(65)

EBV	Mouse	EBV-specific bulk CD4^+^ T cell cultures against a murine model of PTLD	(66)
	Human, *in vitro* model	LMP2a RNA-transfected dendritic cells for the treatment of EBV-positive Hodgkin disease	(67)
	Human, *in vitro* model	EBNA1-specific CD4^+^ T cells against EBV-carrying natural killer and T cell lines from patients with chronic active EBV infection	(68)
	Human, *in vitro* model	EBV latency II-derived peptides (EBNA1, LMP1, and LMP2) against EBV latency II malignancies	(69)
	Human, *in vitro* model	HLA II LMP1-derived candidate peptides vaccination against natural killer lymphoma cells	(70)
	Human, *in vitro* model and mouse	HLA II promiscuous peptide cocktail vaccine against EBV latency II malignancies	(71)

HPV	Human	Long peptide vaccination against HPV-16 for vulvar intraepithelial neoplasia	(72)

*IAV, influenza A virus; CMV, cytomegalovirus; MCMV, murine cytomegalovirus; TCR, T-cell receptor; HIV, human immunodeficiency virus; EBV, Epstein–Barr virus; PTLD, posttransplant lymphoproliferative disease; LMP, latent membrane protein; EBNA, Epstein–Barr nuclear antigen; HPV, human papillomavirus*.

During viral infections, the pivotal role of the immune system, through the synergistic action of both innate and adaptive immune cells, is to prevent, control, and respond to several viropathogenic processes. Specific antiviral immunity has been traditionally associated with the T-cell compartment, and, for long time, direct cytolytic activity against virus-infected cells has been solely assigned to CD8^+^ CTL (73–75).

Historically, cytolytic CD8^+^ T cells have been recognized as the major contributors to the control of acute and chronic viral infections even though, in the last decade, many studies described the presence of activated CD4^+^ T cells in the peripheral blood of HIV-1-, CMV-, and EBV-infected patients (76–78). Indeed, given the fact that CD8^+^ CTL are often unable to efficiently control viral replication, the involvement of alternative immune pathways should not surprise. Moreover, different viral immune escape mechanisms have been described, including the ability to downmodulate the expression of MHC class I molecules recognized by effector CD8^+^ T cells and the generation of mutated CD8^+^ epitopes in the HIV-1 setting (79, 80), where error-prone reverse transcription during viral replication produces “variant” epitopes. As a result, these immune escape mechanisms strongly impair CD8^+^ CTL recognition and promote disease progression (81). Thus, during viral infections, CD4^+^ CTL may act in concert with cytolytic CD8^+^ T cells, bearing thus some advantages in terms of infection control. Indeed, the simultaneous induction of CD4^+^ and CD8^+^ CTL increases the chance of killing virally infected target cells, thanks to the dual recognition through MHC class I and II. Moreover, the nature of the MHC class II structure allows for a greater diversity in the type and number of viral epitopes presented in comparison to MHC class I (82). In addition, several exceptions showed that the presentation of peptides in a MHC class II context is not strictly restricted to exogenous proteins (83, 84). Thus, CD4^+^ CTL play a pivotal role restraining viral infection with tropism for MHC class II-positive target cells, as infected lung alveolar or airway epithelial cells, EBV-transformed B cells, or HIV-1-infected human CD4^+^ T cells (26, 39, 50).

The therapeutic potential of CD4^+^ CTL may be exploited also for vaccination purposes against HIV-1 infection, since these effectors are able to efficiently recognize and kill infected APC like macrophages, which constitute long-lived viral reservoirs (82). Similarly, CD4^+^ CTL may indirectly attenuate influenza virus-induced morbidity through the killing of TNF-inducible nitric oxide synthase DC, the major injury-inducing cells during influenza virus infection, which harbor influenza virus antigen and express high levels of MHC class II (50).

Targeting professional APC also represents a regulatory capacity attributed to CD4^+^ CTL to curtail the immune response, thus avoiding an excessive inflammatory reaction through the priming of naive T cells and the systemic spread of the pathogen (39, 40, 51). In addition, the differentiation in cytotoxic cells prevents CD4^+^ T cells from becoming inflammatory Th1 or Th17 cells, thus limiting the excessive infiltration of systemically activated cytokine secreting cells, which may be responsible for tissue damage (39, 40). The elevated numbers of CD4^+^ CTL observed in chronic inflammatory conditions such as autoimmune and inflammatory diseases suggest their pathogenic role in these conditions; indeed, their amount is related to disease severity (51). However, their precise contribution in the pathogenesis of autoimmunity and inflammatory diseases and the nature of the antigen (self or virus-derived) that drives their specificity are yet to be completely understood (85).

For these reasons, new therapeutic strategies including vaccine approaches aim at the simultaneous induction of cytolytic CD4^+^ and CD8^+^ T cell responses, with the aim to increase the chance of killing virus-infected cells, due to the recognition of a wider spectrum of epitopes presented by both MHC class I and II molecules.

## Influenza Virus Infection

Influenza A virus and, at a lower extent, influenza B viruses are annually responsible for infections of the respiratory tract in about 5 million people worldwide, which can evolve to further complications and death in high-risk groups, including immunocompromised subjects, children, and elderly (86). Although new preventive vaccines are developed every year that renew specific immune responses to influenza virus in infected individuals, the continuous viral evolution still represents an intriguing challenge to the development of highly effective vaccination strategies.

Influenza virus genes code for the two envelope proteins, namely hemagglutinin and neuraminidase, which mediate viral attachment, entry, and release from infected cells, and for several internal proteins involved in viral replication (87). Current vaccines are developed with the main purpose to generate humoral responses against membrane hemagglutinin and neuraminidase (88). These formulations generally contain selected dominant epitopes recognized by antibodies and, to a lesser extent, by specific T cells (89). Notably, individual’s selective immune pressure due to previous infections and due to the high-mutation rate of influenza virus genome, particularly in the antigenic sites of hemagglutinin and neuraminidase (90, 91), gives rise to new viral variants, which become able to evade pre-existing acquired immunity. In this context, humoral responses no longer provide efficient protection against infection with antigenically mismatched virus strains, since less than 3% of epitopes recognized by vaccine-induced antibodies are conserved (89). Conversely, the majority of IAV-specific T cell responses are directed against epitopes located in more conserved viral proteins, such as the internal nucleoprotein (NP) and the matrix (M)-1 protein (92, 93). Therefore, about 15% of IAV-derived T cell epitopes maintain almost identical sequences, rendering these proteins ideal targets for cytolytic T cells (94). On these grounds, in the last years, many efforts have been focused on the development of combined vaccine formulations, able to elicit both antibody and cellular responses and, interestingly, endorsing an even more direct contribution of CD4^+^ CTL to viral clearance and protection from reinfection.

It is well established that CD8^+^ CTLs play a crucial role in the response to influenza infection, as demonstrated in both mouse models and humans (95, 96), while the relationship between influenza-specific CD4^+^ T cells and disease protection and limitation was investigated only in recent years. In this respect, Wilkinson and colleagues recently found a pre-existing memory CD4^+^ T cell population in peripheral blood of healthy volunteers, which was able to recognize and respond to peptides from NP and matrix protein of the influenza virus (97) (Figure 2). Moreover, phenotyping analysis showed that these cells may have cytolytic characteristics, as they stain positive for CD107a and actively produce IFNγ upon antigenic stimulation with influenza protein peptide pools (97), thus supporting their cytotoxic activity. These results were also in line with previous findings showing an increased frequency of CD4^+^ CTL in the circulation of healthy donors during viral infection (6). Further *in vivo* studies elegantly conducted by Hua et al. demonstrate that CD4^+^ T cells with cytotoxic potential are specifically induced at the site of infection during acute respiratory influenza (50). In particular, they examined the expression of granzyme B, perforin, and IFNγ in CD4^+^ T cells from the lung and in draining mediastinal lymph nodes, showing the presence of cytolytic effector cells only at the site of infection, as further confirmed by the upregulation of CD107a in the same cells (Figure 2). Accordingly, a recent study demonstrated that granzyme B- and perforin-producing CD4^+^ T cells were able to recognize and kill influenza virus-infected cells at the site of infection (98). In addition, the authors found that the cooperation between IFN type I and IL-2 pathways drives the development of CD4^+^ T cells with cytotoxic potential *in vivo*, mainly through the action of T-bet and Blimp-1 (50). Therefore, taking together, these data suggest that the presence of CD4^+^ CTL responses at the site of infection may limit virus shedding, replication, and illness severity. The evidence of pre-existing CD4^+^ T cell populations to influenza virus and their direct involvement in the *in vivo* elimination of virus-infected cells during primary and secondary infections have prompted Savic and colleagues to the development of an epitope-based universal influenza vaccine (60) (Table 1). In their study, the researchers identified a library of IAV-derived CD8^+^ and CD4^+^ T-cell epitopes. Selected panels were then validated by monitoring T cell responses in a cohort of healthy donors, and high levels of specific responses were achieved when PBMCs were stimulated with core protein-derived epitopes. As expected, the lowest detected responses were among the CD8^+^ T cells stimulated to the external viral protein-derived peptides. More interestingly, functional analysis revealed that the CD4^+^ T cell compartment was dominated by cells producing IFNγ/IL-2/TNF-α after stimulation with epitopes derived from either internal or external viral proteins, while CD8^+^ T cells were almost all single cytokine producers (60). This is particularly relevant since it has been demonstrated that triple cytokine-producing CD4^+^ T cells are functionally superior not only for their costimulatory and degranulation potential, which plays a crucial role in controlling infection by conferring protection from influenza through perforin-mediated cytotoxicity (99), but also for the establishment of durable memory T cell responses (100). Interesting insights in the context of enhancing influenza virus vaccine strategies came from new understandings on the intrinsic mechanisms by which specific CD4^+^ effectors may lead to the generation of effective CD4^+^ memory T cells. In this regard, Devarajan and colleagues have recently hypothesized a two-step vaccination approach that elicits effective CD4^+^ cytotoxic responses, by initially inducing a strong and proficient activation of APC and later providing antigens to establish long-lasting cellular immunity (101). In particular, it was demonstrated that the peculiar inflammatory microenvironment of the respiratory epithelium during influenza virus infection strongly promotes the activation of APC secreting high levels of IL-6 and drives optimal T-cell priming directly at the site of infection (54, 98). In this context, the costimulation with CD8^+^ T cells and IL-2 signaling occurring few days after initial priming may drive the efficient transition of these CD4^+^ effectors to memory cells, thus ensuring a long-lasting protection from secondary infections (101, 102). On these grounds, the authors emphasized the need to induce both multifunctional CD4^+^ and cytotoxic CD8^+^ T cell responses to IAV, to produce optimized universal vaccines.

## CMV Infection

Cytomegalovirus is a herpes virus that infects the majority of the human population, establishing a lifelong and largely asymptomatic infection in immunocompetent people, while conversely causing severe disease in immunocompromised hosts (103). CMV can mediate direct effects when the virus is detected in patient’s peripheral blood or in organ biopsies and establishes the so-called CMV syndrome characterized by severe inflammatory status due to widespread tissue invasion (104).

During primary infection, the adaptive immune system plays a pivotal role in fighting virus replication, being able to control CMV latency after infection resolution (105). Interestingly, recent data demonstrated that the development of specific CD4^+^ T cell responses is tightly related to both latent and chronic CMV infection (106) and is crucial for protection against this virus (107). During CMV latent infection, CD4^+^ T cells are characterized by a peculiar CD27^−^/CD28^−^ phenotype (108), the loss of IL-7Rα, expression of CD57 and KLRG1, and a decreased proliferative capacity, suggestive of a cytotoxic CD8^+^ T cells-like phenotype (109, 110). Indeed, increasing evidence suggests that CD4^+^ T cells are involved in the control of CMV infection through a direct cytotoxic activity (82), which was even found to precede CMV-specific antibody and CD8^+^ T cell responses in asymptomatic patients (107). Moreover, data showing virus persistence in the salivary glands of murine CMV (MCMV)-infected mice upon the depletion of MCMV-specific CD4^+^ T cells further confirmed their central role in antiviral control (111). The relevance of CD4^+^ CTL at the sites of CMV infection was argued in pioneering work by van Leeuwen et al., in which the authors described that the expansion of CD4^+^CD28^+^ T cells was driven by the decrease of CMV viral load occurring early after primary infection (112). Furthermore, this CD4^+^ effector cell subpopulation was characterized by the expression of granzyme B cytotoxic granules, whose production appeared further increased in CMV-seropositive renal transplant recipients possibly due to the chronic exposure to the virus (111).

Interesting insights about the acquisition of cytolytic potential by CD4^+^ T lymphocytes come from Casazza and colleagues, who investigated the contribution of these cells to the control of viral replication in a cohort of CMV-seropositive healthy donors (78). They detected strong and specific responses to CMV pp65-derived epitopes in almost all analyzed individuals. More interestingly, the frequency of CMV-specific CD4^+^ T cells appeared to be higher than those required to solely ensure the production of antibodies and functional CD8^+^ T cells, supporting their direct cytotoxic effect in antiviral response. Accordingly, through extensive phenotype analysis, the authors demonstrated that these effector cells produce granzyme A and B, perforin, MIP-1β, TNF-α, and IFNγ, even though no evidence of a direct link between surface mobilization of CD107a, perforin, and granzyme content and killing was observed (78). Similar results were also obtained by Chiu et al., who reported a polyfunctional phenotype for CMV-pp65-specific CD8^+^ and CD4^+^ T cells and higher CMV-specific IgG serum levels in association with cytotoxic activity and maturation of both cell subtypes during aging (113). Furthermore, it is known that CMV glycoprotein B (gB) is efficiently presented by MHC class II molecules (114) and is one of the major candidates for a subunit vaccine approach (115) (Figure 2). In this regard, an interesting paper described the isolation of several gB-specific CD4^+^ T cell clones directed against three HLA-DR- and -DQ-restricted peptides from chronically CMV-infected individuals (61) (Table 1). Accordingly, Pachnio et al. found that gB-specific CD4^+^ T cell responses are characterized by a high production of IFNγ, TNF-α, and granzyme B (116) (Figure 2).

Among the latest epitope-based vaccination strategies in the CMV setting, relevant data emerged from the work of Benedict and colleagues, who explored the role of CD4^+^ CTLs in the context of virus-infected mice. They found that only 2 of 234 screened epitopes were able to activate CD4^+^ T cells, produce high levels of granzyme B, and kill target cells. Moreover, the authors demonstrated that the vaccination of immunocompetent mice with the two selected peptides significantly reduced MCMV replication and provided protection from virus reinfection (62) (Table 1). More interestingly, direct CD4^+^ cytolytic activity was also observed in some organs (62). These results were also confirmed by Jeitziner et al., in which the authors generated TCR transgenic mice that are able to recognize a MCMV-specific CD4^+^ T cell epitope within the M25 protein (63) (Table 1). In particular, by analyzing virus-specific effector cells isolated during primary MCMV infection, they identified a monoclonal CD4^+^ T cell subpopulation exerting antiviral protective functions through an IFNγ-dependent mechanism. A recent study by Pachnio and colleagues demonstrated that chronically CMV-infected individuals present moderate percentages of virus-specific CD4^+^ T cells, increased expression of NKG2D, and low levels of PD-1. Finally, transcriptional profiling analysis highlighted a significant upregulation of cytolytic specific genes, in particular granzymes and perforin, and a distinctive secretome signature in virus-specific CD4^+^ T cells (117).

## HIV Infection

Human immunodeficiency virus 1 is essentially an infection of the immune system, which almost invariably results in an irreversible state of immunodeficiency (namely acquired immune deficiency syndrome) when left untreated. HIV-1 pathogenesis is characterized by the progressive infection and depletion of CD4^+^ T lymphocytes that normally coordinate the adaptive T and B cell response to defend the host from intracellular pathogens. Indeed, there were early signs that, in the acute HIV-1 infection, up to 60% of all activated memory CD4^+^ T cells are infected and subsequently depleted from all tissue compartments (118). Many studies well documented that HIV-1-specific CD8^+^ T cells are strong contributors to the control of viral replication and disease progression. In particular, robust and highly specific CD8^+^ T cell responses against HIV-1 have been long observed in infected subjects (119, 120), and the same effectors are also able to inhibit HIV-1 replication (121). Unfortunately, even though CD8^+^ T cell responses clearly occur during the acute phase of infection (122), the major responses are directed to epitopes within envelope proteins, which are among the most variable in the virus (123, 124). Consequently, escape mutations in targeted epitopes can significantly impair CD8^+^ T cell ability to recognize and kill HIV-1-infected cells (125). Moreover, some acute phase epitopes may not be those targeted in chronic infection, when proteins may be novel or their function may be lost, as observed with the upregulation of both negative immunoregulatory molecules, such as PD-1, and transcription factors inhibiting cell proliferation and cytokine release (126, 127). Conversely, the peptide-binding modalities of MHC class II that characterize CD4^+^ T cells allow for much greater sequence diversity while maintaining the affinity of peptide-MHC interaction and epitope recognition by TCR, thus decreasing the ability of the virus to escape from HIV-1-specific CD4^+^ T cell responses (128). As a matter of fact, even though MHC class II epitopes may also be prone to immune escape (129), this phenomenon is less frequently observed.

On these grounds, growing evidence suggests that induction of cytotoxic CD4^+^ T cell responses might be relevant for the successful control of HIV-1 infection and for the development of highly efficient anti-HIV-1 vaccines (65) (Table 1). Recently, Johnson and colleagues demonstrated that HIV-1-specific cytolytic CD4^+^ T cells have a distinct transcriptional and phenotypic signature compared to Th1 CD4^+^ cells (130). In particular, by analyzing the transcriptional profile of Gag-specific CD107a^+^ IFNγ^+^ CD4^+^ T cells with unsupervised hierarchical clustering, they found cytolytic effector features similar to HIV-1-specific CD8^+^ CTLs and NK cells. In contrast, Gag-specific Th1 CD4^+^ T cells displayed higher expression of several surface markers associated with helper CD4^+^ T cell functions. Moreover, the authors reported that surface expression of CD57^+^ in cytolytic CD4^+^ T cells occurs early during acute HIV-1 infection, suggesting their involvement in the initial stages of HIV-1 acquisition (130, 131). Meanwhile, relevant data obtained by Rosenberg et al. supported the idea that the persistence of a perforin-positive CD4^+^ T cell population into all stages of chronic disease progression might be associated with a better control of viral replication (132, 133). On the other hand, subsequent studies clearly demonstrated that high levels of HIV-1 Gag-specific CD4^+^ CTL could be detected in long-term non-progressors successfully controlling their infection (8, 134). Extensive analysis of HIV-1-specific CD4^+^ T cell responses revealed that, even though HIV-1 is characterized by a high selection pressure, immunodominant epitopes recognized by these effectors are mainly derived from Gag and Nef proteins (135), known to be mostly conserved along the natural history of infection. Accordingly, *in vivo* studies conducted by Sacha and colleagues demonstrated that macaques able to control SIV infection were characterized by strong Gag- and Nef-specific CD4^+^ T cell responses (136). Notably, after *in vitro* long-term culture, these CD4^+^ T cells become unable to recognize and kill infected CD4^+^ T lymphocytes, while they still suppressed viral replication in long-lived reservoirs, especially macrophages (136–138), which can be thus considered as potential vaccine targets for these effectors (Figure 2). In this regard, many efforts have been spent in the last years to design prophylactic vaccine against HIV-1 that, beside specific CD8^+^, are also able to elicit effective CD4^+^ T cell responses. However, the use of CD4^+^ CTL-based vaccine approaches in the HIV-1 setting still represents an unsolved issue. The main skepticism derives from the idea that the activation and expansion of these cells could represent a double-edged sword, since it is well known that resting CD4^+^ T cells are preferential HIV-1 reservoirs. Indeed, the antigen-specific CD4^+^ T cell response developing during primary HIV-1 infection includes a high percentage of CD4^+^ CTL, which express CCR5 and therefore may be highly susceptible to HIV-1 infection (8, 139). Moreover, vaccine-induced CD107a^+^ CD4^+^ T cells are selectively depleted following virus infection (64) (Table 1). Nevertheless, the only vaccination trial that recently showed some level of protection was the Phase III RV144 Thai trial (65) (Table 1). This vaccine showed moderate protection against HIV-1 infection and, more interestingly, revealed that most vaccinated HIV-negative individuals presented predominantly polyfunctional effector CD4^+^ T cell responses against the V2 region of the envelope protein (140, 141).

## Epstein-Barr Virus Infection

Epstein–Barr virus is a human γ-herpesvirus associated with the development of different forms of B cell and epithelial malignancies, which account for 1% of the total human cancers (142). In healthy and infected individuals, the life cycle of this virus is characterized by a continuously evolving relationship with host immune system. Indeed, EBV is a highly immunogenic virus as demonstrated by the strong response elicited at the time of primary infection, which successfully constrains the virus in a strictly latent, immunologically silent status, where only latent membrane protein (LMP) 2 and/or Epstein–Barr nuclear antigen (EBNA) 1 proteins are expressed. The immune system is then deputed lifelong to control the occasional cycles of “reactivation from latency-viral particle production-reinfection” and also to restrain the oncogenic potential of the virus. Indeed, the emergence of EBV-related tumors is invariably linked with overt and systemic or more subtle and local impairments of the immune system (143). Therefore, to guarantee its own persistence and establish latency in memory B cells, EBV has evolved different strategies to evade both CD8^+^ and CD4^+^ T cell recognition (144). Besides the shutting down of the most immunogenic latent proteins and the production of the viral IL-10 homolog encoded by the *BCRF-1* gene, the expression of the lytic proteins BNLF2a, BILF1, and BGLF5 strongly impairs HLA class I presentation pathway, while the recognition by CD4^+^ T cells is targeted by the glycoprotein gp42, the lytic cycle inducer BZLF1 (145), and multiple miRNA (146). In particular, such miRNA display their action immediately after B cell infection by reducing the differentiation of naive CD4^+^ T cells into Th1 cells and the activation of CD4^+^ CTL effectors. These very recent findings emphasize the importance of the CD4^+^ T cell arm in the complex interplay between the virus and the host immune response. Indeed, while numerically subdominant (CD4^+^ T cell frequency is 10-fold lower than that of CD8^+^ T cells) (147), EBV-specific CD4^+^ T cells complement CD8^+^ T cell responses in terms of both lytic and latent antigen recognition and kinetics. Indeed, they respond to all classes of lytic antigens (148), while CD8^+^ T cells neglect late lytic antigens due to the action of immunoevasins. As regard latent proteins, CD4^+^ T cells preferentially recognize EBNA1 and LMP proteins (Figure 2), which provide only subdominant antigens, if any, to CD8^+^ T cells. Moreover, possibly as a result of the preferential antigen feeding pathway (149) that relies on the mature protein pool rather than newly synthesized proteins, CD4^+^ T cells recognition is quite delayed, but persistent.

In addition to the well-accepted helper activity, the potential importance of CD4^+^ CTL is suggested by their presence in EBV carriers, as demonstrated directly *ex vivo* by Appay et al. (6). Even during infectious mononucleosis, circulating granzyme B^+^ CD4^+^ T cell are detected in the blood, and in sharp contrast with the CD8^+^ T cell compartment, their number does not correlate with symptom severity, thus suggesting a potential protective role (150). However, CD4^+^ T cells isolated from tonsils where EBV infection of naive B cells occurs are not directly cytotoxic but acquire this potential only *in vitro* (151). The *in vivo* relevance of CD4^+^ CTL in the different phases of infection and in tumor development is rarely directly demonstrated and can be principally inferred from *in vitro* experiments as well as from clinical results of immunotherapeutic interventions. Indeed, the majority of data relies on *in vitro* expansion of EBV-specific T cells from PBMC of EBV-seropositive healthy donors, and the lytic activity is demonstrated in both short-term standard chromium release assays and long-term outgrowth inhibition studies. The cytotoxic potential of such CD4^+^ T cells appears to be mediated by the release of cytotoxic molecules, such as perforin, granzyme, and granulysin (66, 152), or by Fas/FasL interaction (153, 154), as demonstrated with the use of selective inhibitors of each pathway (Figure 2). Even long-term growth inhibition of target cells, which is more suggestive of an *in vivo* situation, depends on lytic activity: indeed, in most cases, it is insensitive to cyclosporin A, which blocks cytokine secretion that requires NFAT-dependent gene transcription, while leaving unaffected the cytotoxic potential.

CD4^+^ T cells endowed with cytolytic capacity have been described against both lytic and latent antigens. With regard to the former, immediate early, early, and late lytic phase antigens are equally recognized by these effectors. These antigens gain access to the MHC class II processing and presentation pathway primarily through receptor-mediated uptake (155–157). Indeed, release of virions even from few tumor cells undergoing lytic cycle can sensitize to killing neighboring cells with a very high efficiency (less than 1 virion/cell can induce recognition of target cells) (156). In particular, lymphoblastoid cell lines (LCL) can be regarded as a model for foci of replication in posttransplant lymphoproliferative disease (PTLD). Therefore, T cells specific for lytic phase antigens can limit the viral spreading and the *de novo* infection in healthy host and EBV-seronegative recipients of transplants from EBV-seropositive donors. In an adoptive immunotherapy setting, their efficacy can be increased with the concomitant use of lytic cycle inducers, like epigenetic and chemotherapeutic agents (158).

Among latency proteins, attention was primarily focused on CD4^+^ T cell responses to EBNA1, since this protein for a long time was regarded as a silent CD8^+^ T cell target (159) (Figure 2). This protein is expressed by all EBV-related malignancies and is the unique viral protein in type I latency tumors, like Burkitt lymphoma (BL). Interestingly, in EBV-positive healthy donors, a CD4^+^ T cell response is constantly detected, and it is strongly skewed toward a Th1 phenotype, as assessed by the cytokine-release pattern and by the presence of IgG_1_ EBNA1-specific antibodies (160, 161). Moreover, BL cell lines can be an *in vitro* target of the cytotoxic activity of EBNA1-specific CD4^+^ T cells (162), and a BL tumor in a mouse model could be eradicated in the total absence of CD8^+^ T lymphocytes, although without involving a direct cytotoxic activity (163). EBNA1-specific CD4^+^ T lymphocytes seem to have an important protective role *in vivo*, as inferred by their reduction or absence in PTLD patients (164), in some pediatric forms of BL (165), in EBV-related Hodgkin lymphoma (166), in NHL that develop in HIV patients (167), and in central nervous system lymphomas (168). Interestingly, Heller et al. (166) suggested that a delayed/reduced presence of EBNA1-specific antibody, as well as CD4^+^ T cells, observed in infectious mononucleosis patients (169, 170) can be one of the possible explanations for the increased risk to develop Hodgkin lymphoma after symptomatic acquisition of EBV infection. Conversely, in multiple sclerosis patients, a relevant presence of EBNA1-specific CD4^+^ T cells, partly cross-reacting with myelin antigens (171), has been recently described, even though a direct pathogenic role for cytotoxic activity of these cells has not been demonstrated yet either in multiple sclerosis (172) or other autoimmune diseases (173, 174).

Other valuable targets of CD4^+^ T cell activity are represented by LMPs, LMP1 and LMP2 (77), and EBNA2 (175), due to their pivotal role in persistence and malignancy (Figure 2). In particular, EBNA2 is one of the first proteins produced after infection and before immortalization, and it is a transcriptional factor required for the initiation and maintenance of B cell growth and transformation. In both cases, cytotoxicity displayed by CD4^+^ T cells is the key effector mechanism that restrains LCL outgrowth and B cell proliferation induced by EBV.

These encouraging preclinical data, and the results of a phase I/II clinical trial (176) demonstrating a correlation between the percentage of CD4^+^ T cells in the infusates and the clinical outcome, prompted the study of new protocols (67–69, 177) and the definition of MHC class II-restricted epitopes (70, 71, 178, 179) to target with improved efficacy of distinct viral proteins for immunotherapeutic purposes (Table 1). These are of great value in the context of EBV-related tumors, which express a limited set of viral proteins (latency II and possibly latency I malignancies) and are characterized by a reduced immunogenicity; moreover, they may also contribute to solve the matter of specificity found using the classical protocol based on reactivation of EBV-specific T cells by LCL restimulation. As advanced by Long et al. (180), in addition to lytic and latent antigens, CD4^+^ T cells could also target B cell-associated antigens. It is worth noting that they generated CD4^+^ T cells through restimulation with mini-LCL instead of LCL: this excludes the reactivation of lytic cycle-specific T cells and potentially biases and overestimates the selection of autoantigen-specific T cells. By the way, the infusion of bulk cultures containing up to 98% of CD4^+^ T cells of undefined specificity was carried out in patients without adverse events, thus indicating that such B cell-specific T cells, if present, likely target an antigen(s) overexpressed by tumor cells (176).

The use of polyfunctional CD4^+^ T lymphocytes that potentially exert both helper and effector functions should be hence promoted in fighting EBV-associated malignancies, but not without keeping in mind some potential drawbacks. In particular, a lytic activity mediated by the Fas/FasL interaction could be responsible for a bystander killing (181), which can potentially damage the tumor-surrounding tissues. Moreover, some epitopes appeared to preferentially elicit CD4^+^ T cells endowed with regulatory functions, as described for some LMP1 and EBNA1-derived epitopes (182). This might be detrimental and harmful, since it could turn off the activity of the infused and autologous T cells. Moreover, a subset of CD4^+^ T lymphocytes may sustain the primary infection of B lymphocytes or even induce the expansion of tumor B cells mainly through IL-4 and IL-13 release and CD40 engagement, as demonstrated *in vitro* (183) and *in vivo* in hu/SCID mouse models (184). However, the B cell help activity seems to be exerted particularly by CD4^+^ T lymphocytes with a Th2 pattern of cytokine release and poor cytolytic activity.

Anyway, with few exceptions, e.g., the *in vivo* model involving the murine γHV68 infection in which the cytotoxic activity of M2-specific CD4^+^ T cells was demonstrated by *in vivo* cytotoxicity assay (185), the demonstration of *in vivo* lytic functions exerted by CD4^+^ T cells can be achieved only through indirect evidence, as previously reported by our group (66). By exploiting a clinically relevant protocol involving PBMC restimulation with LCL, we generated EBV-specific bulk CD4^+^ T cell cultures characterized by an *in vitro* cytotoxic activity and a significant *in vivo* antitumor effect against a murine model of PTLD (Table 1). This result was apparently achieved without the cytokine-mediated recruitment of other effector cells, since neither the exogenous administration nor the blocking of CD8^+^ T cell-secreted IFNγ had any impact on LCL biology. Conversely, the therapeutic activity of CD4^+^ T cells appeared to critically depend on HLA class II-mediated interaction with target cells. Indeed, the decitabine-mediated partial recovery of HLA class II downmodulation that characterizes LCL upon *in vivo* inoculation improved LCL recognition by CD4^+^ T cells and prolonged survival in drug-treated mice. Overall, the downmodulation of HLA class II is apparently limited only to the mouse model, as HLA-DR is consistently expressed by tumor cells at all stages of disease in human PTLD specimens (66). Therefore, in line of principle, CD4^+^ T cells could be efficiently administered to PTLD patients without any additional pharmacological treatment.

## Hepatitis B and C Viruses

Hepatitis B virus (HBV) and HCV are two hepatotropic, non-cytopathic viruses, classified by the International Agency for Research on Cancer as carcinogens of Group 1 to humans (186). Indeed, chronic infection with HBV and HCV represents a major risk for the development of cirrhosis and finally hepatocellular carcinoma (HCC). In the long path from acute infection to overt tumor, which can take several decades, the cellular immune response appears to be a double-edged sword accounting for the clearance of the virus and even the spontaneous regression of HCC (187), but also for the extent of liver cell damage when the infection cannot be resolved. In the tolerogenic milieu of the liver (188), the functional state of tumor infiltrating lymphocytes and in particular the balance between Treg and CTL (as assessed by Foxp3 and granzyme B immunohistochemical staining of tumor specimens) significantly impact the prognosis of HCC cancer patients in terms of 5-year overall survival and disease-free survival (189). In this complex picture, the relevance of dissecting the potential role of CD4^+^ T cells is suggested by the sustained expression of HLA class II molecules in HBV-infected cells (190) and HCC and by the association of HLA-DR13 expression with a self-limited course of HBV infection (191–193). In particular, the protection mediated by HLA-DR13 molecule is linked to the effective presentation of hepatitis B core antigen (HBcAg) and the consequent induction of a vigorous proliferative antigen-specific CD4^+^ T cell response (194) (Figure 2). It is commonly held that the role of CD4^+^ Th cells is protective with respect to HBV and HCV infections, as demonstrated in animal models (195, 196) and human beings (197–199). While the protective role of CD4^+^ T cells is consistently associated with a Th1 activity, the role of CD4^+^ CTL still remains controversial. In a cohort of 76 patients with viral hepatitis (15 HBV, 22 HBV/hepatitis D virus, and 17 HCV), the frequency of perforin^+^ CD4^+^ T cells, as assessed directly *ex vivo* by flow cytometry, was highly variable (ranging from less than 1% to more than 25%) but consistently higher than in healthy controls (190). In particular, in HBV/HDV coinfected individuals, this parameter correlates with elevated aspartate aminotransferase levels, diminished platelet count, and fibrogenesis in elderly individuals (190). In line with these findings, the frequency of perforin^+^ CD4^+^ T cells was found to decrease significantly only in two patients who cleared the virus spontaneously. In sharp contrast, Fu et al. (200) demonstrated that in HBV-related HCC, the loss of perforin and granzyme A and B-expressing CD4^+^ CTL was associated with high mortality rate and reduced survival time. Indeed, the number of circulating CD4^+^ CTL was consistently higher in HCC than in chronic HBV carriers and liver cirrhosis patients, but decreased as tumor progressed. Interestingly, based on the notion that responding T cells preferentially compartmentalize into the liver (201), the presence of CD4^+^ CTL was investigated in tumor specimens. Even in this case, the number of this cell subset was found to decrease when moving from cancer stage I to III, and the expression of the degranulation marker CD107a was also significantly reduced in the advanced stages of the disease, as a consequence of the direct action of local Treg cells. As demonstrated by another research group (202), cytotoxic activity of CD4^+^ T cells was also negatively regulated by Tim^+^ B cells through an IL10-mediated mechanism in HCC patients with history of chronic HBV infection. Indeed, these patients were characterized by a negative correlation between IL10-expressing B cells and granzyme A^+^/granzyme B^+^/perforin^+^ CD4^+^ CTL in the periphery. However, it is questioned whether these regulatory B cells suppress a CD4^+^ CTL-mediated effective antitumor response or rather protect the liver from a potential pathogenic role mediated by the same cell subset. While subject to a negative regulatory activity, CD4^+^ CTL themselves can also exert a regulatory role, as suggested by Cao and coworkers (203). They described the generation of HBcAg-specific HLA-DR13-restricted Th1 type CD4^+^ T cell clones endowed with *in vitro* lytic activity, from a subject who recovered from a previous acute HBV infection. Upon *in vivo* transfer in a hu-PBL-NOD/SCID mouse model, these T cell populations induced the drop of anti HBcAg-specific IgG and IgM, as the consequence of the lysis of HBcAg-binding or -specific B cells. This B cell population is detected at high frequency in both human and mice (204, 205), but its beneficial or detrimental role in the natural course of HBV infection remains to be determined. As suggested by the authors (203), if HBcAg-specific (or -binding) B cells and/or the produced antibodies would sustain the persistence of HBV infection, then the elimination of such B cells could promote viral clearance. A more direct therapeutic activity of CD4^+^ CTL was reported in a preclinical model of vaccination (206). The authors describe the suppression of tumor formation in mice vaccinated with DC fused with HCC cells and demonstrate that protection was mediated by the CD4^+^ T cells elicited, since it was completely abrogated by anti-CD4 antibodies. These T cells appeared to be endowed with a robust, albeit non-MHC-II restricted, cytotoxic activity involving the release of cytotoxic granule content. To the best of our knowledge, very few other articles investigated the cytotoxic potential of CD4^+^ T cells induced by vaccination strategies (207) or by *in vitro ad hoc* protocols (208). Indeed, the majority of papers focused the attention on the release of “classic” Th1 cytokines only (209–212), thus possibly underestimating the potential contribution of CD4^+^ CTL to the observed results.

## Human Papillomavirus (HPV)

Human papillomavirus is causally linked with the development of cancer of the anogenital region, mainly due to the presence of the two oncoproteins E6 and E7 that act by inhibiting the tumor suppression proteins p53 and pRb, respectively (213). Despite the numerous immune evasion strategies displayed by the virus, host immune response clears the infection in the majority of infected people (90%) and only a minor fraction of chronically infected individuals ultimately develop cancer (214). An important protective role in HPV clearance and control of its neoplastic consequences is mediated by CD4^+^ T cells (Figure 2). Their presence and fine antigen specificity, as well as their functional properties, have been thoroughly investigated especially in the context of HPV16 and HPV18 infections, the two most widely diffused high-risk genotypes (214). As demonstrated in different reports (72, 215–226) and recapitulated in one of the largest prospective study involving women with HPV16- and 18-related cervical cancer (227), prevention/control of HPV-related malignancies appeared to be mediated in particular by Th1-polarized CD4^+^ T cell responses. On these grounds, it is tempting to speculate a similar role for also CD4^+^ CTL that may likely derive from this Th compartment (54). However, almost all papers neglected to dissect this issue, and the assessment of their cytotoxic activity was far from being routinely performed. To the best of our knowledge, only Facchinetti et al. (228) described the generation of CD4^+^ T cell lines from healthy people, which were endowed with cytolytic activity and recognized a naturally processed, HLA-DR-restricted epitope derived from the E7 protein of HPV-18 (Figure 2). Conversely, Grabowska et al. (221) failed to observe any cytotoxic activity in CD4^+^ T cells directed against E2, E5, E6, and E7 of HPV-16 in their cohort of healthy individuals. Interestingly, Garcia-Chagollan and colleagues (229) described an increased number of circulating NKG2D^+^ CD4^+^ T cells in CIN1 patients, with respect to healthy controls. This particular subset of CD4^+^ T cells was alternatively described to be characterized by cytotoxic and pro-inflammatory properties or regulatory activity (54, 230). In this study, the role of NKG2D^+^ CD4^+^ T cells was not conclusively established; however, the fact that almost all patients cleared their lesions without progressing to more advanced stages of disease at 1 year of follow-up can suggest a beneficial rather than a detrimental effect for these CD4^+^ T cells. Moreover, these patients showed increased levels of IL-15, a cytokine that promotes cytolytic activity of effector cells, in conjunction with a significant reduction of the anti-inflammatory TGF-β (229).

## Conclusion

Although data accumulated so far are progressively increasing our understanding of the complex biology of CD4^+^ T cells, we are still quite far from being completely aware of the role, functions, and potentialities that cytotoxic CD4^+^ T lymphocytes may have *in vivo*. Available evidence supports the notion that these cells do not represent a mere laboratory artifact, but rather constitute a distinct differentiation status of CD4^+^ T lymphocytes. These important advances stimulate further studies focusing more specifically on the phenotypic and functional characterization of cytotoxic CD4^+^ T lymphocytes in various disease settings. A better knowledge of the microenvironmental and cellular factors that critically drive the differentiation of CD4^+^ T cells in cytotoxic effectors may provide new clues on how to manipulate the plasticity of these cells for therapeutic purposes. Of particular relevance will also be the assessment of their *in vivo* contribution to the clearance of infected cells and the control of infection-driven tumors, both as single immune cell subpopulation and as effectors synergizing with CD8^+^ T lymphocytes and innate immunity cells. These studies will allow the rational background for the design of improved vaccines that will be able to better control infectious diseases through the activation of antigen-specific CD4^+^ T cells also endowed with cytotoxic properties. These strategies will be probably more effective in clearing virus-infected cells, due to the exploitation of a broader spectrum of epitopes presented by both MHC class I and II molecules. Boosting the immune responses mediated by cytolytic CD4^+^ T cells may be also of pivotal importance to improve the efficacy of immunotherapy against virus-driven tumors. In this setting, the selection of MHC class II epitopes able to promote the expansion and activity of CD4^+^ CTL may be helpful to optimize the set of viral antigens targeted by immunotherapeutic approaches. In perspective, therefore, CD4^+^ cytolytic effectors may become integral part of new therapeutic strategies for viral infections and virus-driven tumors.

## Author Contributions

EM, AM, DM, and MC were involved in study design, wrote the first draft of the manuscript, conducted the literature search, reviewed the abstracts, and contributed to the final draft; SDS conducted literature search and revised the manuscript. AR and RD supervised the study and revised the final draft. All authors have read and approved the final manuscript.

## Conflict of Interest Statement

The authors declare that the research was conducted in the absence of any commercial or financial relationships that could be construed as a potential conflict of interest. The reviewer CMLM and handling Editor declared their shared affiliation, and the handling Editor states that the process nevertheless met the standards of a fair and objective review.

## Abbreviations

APC, antigen-presenting cells; CMV, cytomegalovirus; CTL, cytotoxic T lymphocytes; EBV, Epstein–Barr virus; gB, glycoprotein B; HBV, hepatitis B virus; HBcAg, hepatitis B core antigen; HCC, hepatocellular carcinoma; HCV, hepatitis C virus; HIV, human immunodeficiency virus; HPV, human papillomavirus; IAV, influenza A virus; LCL, lymphoblastoid cell lines; LCMV, lymphocytic choriomeningitis virus; MCMV, murine cytomegalovirus; miRNA, microRNA; PTLD, posttransplant lymphoproliferative disease; Runx3, Runt-related transcription factor 3; TCR, T-cell receptor; Th, T helper; ThPOK, Th-inducing BTB/POZ domain-containing Kruppel-like zinc-finger transcription factor; Treg, regulatory T cells.
